# Degradation of phenylethanoid glycosides in *Osmanthus fragrans* Lour. flowers and its effect on anti-hypoxia activity

**DOI:** 10.1038/s41598-017-10411-0

**Published:** 2017-08-30

**Authors:** Fei Zhou, Yajing Zhao, Maiquan Li, Tao Xu, Liuquan Zhang, Baiyi Lu, Xiaodan Wu, Zhiwei Ge

**Affiliations:** 1 National Engineering Laboratory of Intelligent Food Technology and Equipment, Key Laboratory for Agro-Products Postharvest Handling of Ministry of Agriculture, Key Laboratory for Agro-Products Nutritional Evaluation of Ministry of Agriculture, Zhejiang Key Laboratory for Agro-Food Processing, Fuli Institute of Food Science, College of Biosystems Engineering and Food Science, Zhejiang University, Hangzhou, 310058 China; 2 Analysis Center of Agrobiology and Environmental Sciences, Zhejiang University, Hangzhou, 310058 China

## Abstract

This study was aimed at investigating the chemical stability (the thermal, light and pH stability) of phenylethanoid glycosides (PhGs) in *Osmanthus fragrans* Lour. flowers, identifying the degradation products of acteoside and salidroside (major PhGs in *O. fragrans* flowers) by UPLC–QTOF–MS and studying the anti-hypoxia activity of PhGs after degradation. The degradation of PhGs followed first-order reaction kinetics, and the rate constant of acteoside (4.3 to 203.4 × 10^−3^ day^−1^) was higher than that of salidroside (3.9 to 33.3 × 10^−3^ day^−1^) in *O. fragrans* flowers. Salidroside was mainly hydrolyzed to tyrosol during storage, and the degradation products of acteoside were verbasoside, caffeic acid, isoacteoside, etc. In a model of cobalt chloride (CoCl_2_)-induced hypoxia in PC12 cells, the anti-hypoxia ability of PhGs decreased after degradation, which resulted from the reduction of PhGs contents. Particularly, caffeic acid exhibited stronger anti-hypoxia ability than acteoside and could slightly increase the anti-hypoxia ability of degraded acteoside. The results revealed that high temperature, high pH and light exposure caused PhGs degradation, and thus the anti-hypoxia ability of PhGs reduced.

## Introduction

*Osmanthus fragrans* (Thunb.) Lour. (sweet osmanthus), belonging to the Oleaceae family^1^, is widely planted in south and middle China^2, 3^. *Osmanthus fragrans* is famous for its fragrant flowers, which are small flowers with four-lobed corolla and varies in colors, such as white, pale yellow, yellow and orange-yellow^3^. They are widely cultivated as ornamental plants^1, 4^. In China, *O. fragrans* flowers have been used as foods (such as beverage, pastry, paste, etc.)^5, 6^ and traditional Chinese medicine^1^ for a long time. In recent studies, the extracts of *O. fragrans* flowers have showed the abilities of neuroprotection^7^, anti-aging^8^, anti-inflammatory^2, 9^, antioxidantion^10–13^ and inhibiting melanogenesis^10^.

Previous studies revealed that the anti-aging and antioxidant activities of *O. fragrans* flowers were well correlated with phenylethanoid glycosides (PhGs), especially the acteoside (also named verbascoside)^8, 12, 13^. PhGs are a class of naturally occurring phenols, which are abundant in *O. fragrans* flowers with the total phenylethanoid glycoside (TPG) content ranged from 92.66 to 130.57 milligrams of acteoside equivalents (AE) per gram of dry weight (mg AE /g DW)^12^. Acteoside and salidroside were two main PhGs in *O. fragrans* flowers with the content of 32.78 to 71.79 mg/g DW and 4.72 to 16.08 mg/g DW^12^, respectively. Acteoside has the ability of protecting nerve cells, for instance, acteoside protects pheochromocytoma (PC12) neuronal cells against 1-methyl-4-phenylpyridinium ion (MPP^+^)-induced apoptotic or necrotic death^14^. Also acteoside can protect the human neuroblastoma SH-SY5Y cells from *β*-amyloid-induced cell damage^15^ and MPP^+^-induced injury^16^. Salidroside shows similar neuroprotective effects^17–19^. Particularly, salidroside can protect PC12 cell from CoCl_2_-induced hypoxia damage^20, 21^. Thus, we consider acteoside and PhGs in *O. fragrans* flowers have the potential ability of anti-hypoxia in PC12 cell.

Considering the benefits mentioned above, PhGs in *O. fragrans* flowers have potential to be used in functional food or medicine. However, acteoside in solution is unstable, and is easy to be destroyed by a number of factors such as temperature^22, 23^, pH^24, 25^ and light^23, 25^ during storage. There is no more available information in previous literatures on the degradation kinetics and degradation products of acteoside and salidroside.

Therefore, the objectives of this work were (1) to evaluate the effects of temperature, pH and light on the degradation kinetics of acteoside, salidroside and TPG in *O. fragrans* flower extracts (OFE); (2) to identify the degradation products of acteoside and salidroside by UPLC–QTOF–MS/MS; (3) to investigate the anti-hypoxia activities of acteoside, salidroside and TPG after degradation.

## Results and Discussion

### Degradation kinetics of phenylethanoid glycosides

**Figure 1:**
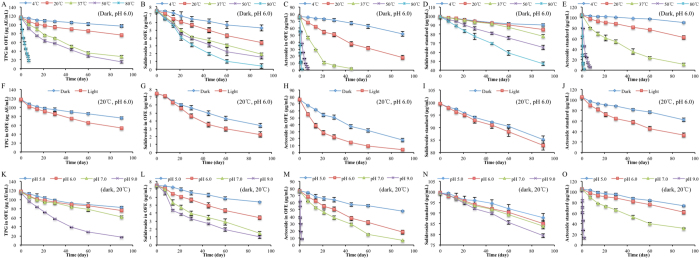
Degradation of phenylethanoid glycosides under different conditions. The effect of temperature on the contents of TPG in OFE (**A**), salidroside in OFE (**B**), acteoside in OFE (**C**), salidroside standard (**D**) and acteoside standard (**E**) at pH 6.0 in the dark; the effect of light on the contents of TPG in OFE (**F**), salidroside in OFE (**G**), acteoside in OFE (**H**), salidroside standard (**I**) and acteoside standard (**J**) at pH 6.0 at 20 °C; the effect of pH on the contents of TPG in OFE (**K**), salidroside in OFE (**L**), acteoside in OFE (**M**), salidroside standard (**N**) and acteoside standard (**O**) at 20 °C in the dark. TPG, total phenylethanoid glycoside; OFE, *O. fragrans* var. *thunbergii* flower extracts.

As shown in Fig. 1A,B and C (contents at 0 day), the TPG, salidroside and acteoside contents in OFE were 117.23 μg AE/mL, 7.46 μg/mL and 76.61 μg/mL, respectively. The acteoside content was slightly higher than that determined by Jiang *et al*.^12^, which might be due to the different origin of *O. fragrans* flowers and/or extraction conditions (solvent, 80% aqueous acetone vs 95% ethanol; temperature, 40 °C vs 20 °C; solid: solvent ratio, 1:15 vs 1:10). For thermal stability of TPG, the contents (after 90 days) decreased by 17.64%, 35.39%, 76.90% and 87.00% at 4, 20, 37 and 50 °C, respectively. As for 80 °C, the TPG content decreased by 84.25% after 7 days. It showed that the degradation of TPG accelerated with the elevation of temperatures. Besides temperature, light and pH also affected the TPG stability, and the degradation of TPG increased with light exposure and the elevation of pH values. The degradation of salidroside and acteoside exhibited similar pattern.

The degradation of PhGs fitted to the first-order kinetic equation with *R* ^2^ higher than 0.94 for all treatments (Table 1). With temperature increasing, the first-order reaction rate constant (*k*) values increased rapidly and the half-life time (*t*_*1/2*_) values decreased (Table 1). It revealed that greater degradation of PhGs occurred at higher temperature. At the same temperature (20 °C), the *t*_*1/2*_ value of PhGs in the light was lower than that in the dark, indicating PhGs were less stable in the light than those in the dark. The pH level also influenced the stability of PhGs. Considering the pH of some products, tea beverages are generally 5 to 7^26^, while detergents such as soap can reach 9. Therefore, pH was set from 5 to 9 in present study. As shown in Table 1, the increase of pH (from 5.0 to 9.0) hastened the PhGs degradation.

**Table 1:** The *k*, *t*_1/2_ and *Ea* values of phenylethanoid glycosides degradation under different conditions.

PhGs	Parameter	T/°C (dark, pH 6.0)					Light (20 °C, pH 6.0)		pH (dark, 20 °C)			
		4 °C	20°C	37 °C	50 °C	80 °C	Dark	Light	pH 5.0	pH 6.0	pH 7.0	pH 9.0
TPG in OFE	*k* (day^−1^)	0.0021 (0.9615)	0.0044 (0.9474)	0.0169 (0.9815)	0.0216 (0.9924)	0.2510 (0.9928)	0.0044 (0.9474)	0.0083 (0.9847)	0.0039 (0.9580)	0.0044 (0.9474)	0.0065 (0.9829)	0.0216 (0.9926)
	*t*_*1/2*_ (day)	330.1	157.5	41.0	32.1	2.8	157.5	83.5	177.7	157.5	106.6	32.1
	*Ea* (kJ/mol)	50.40 (0.9636)										
Salidroside in OFE	*k* (day^−1^)	0.0042 (0.9773)	0.0088 (0.9963)	0.0148 (0.9863)	0.0181 (0.9878)	0.0333 (0.9956)	0.0088 (0.9963)	0.0143 (0.9734)	0.0039 (0.9823)	0.0088 (0.9963)	0.0173 (0.9802)	0.0214 (0.9774)
	*t*_*1/2*_ (day)	165.0	78.8	46.8	38.3	20.8	78.8	48.5	177.7	78.8	40.1	32.4
	*Ea* (kJ/mol)	21.63 (0.9796)										
Acteoside in OFE	*k* (day^−1^)	0.0043 (0.9575)	0.0157 (0.9950)	0.0811 (0.9898)	0.4786 (0.9828)	2.034 (0.9926)	0.0157 (0.9950)	0.0323 (0.9875)	0.0051 (0.9794)	0.0157 (0.9950)	0.0269 (0.9864)	1.1196 (0.9826)
	*t*_*1/2*_ (day)	161.2	44.1	8.5	1.4	0.3	44.1	21.5	135.9	44.1	25.8	0.6
	*Ea* (kJ/mol)	69.14 (0.9829)										
Salidroside Standard	*k* (day^−1^)	0.0012 (0.9618)	0.0017 (0.9945)	0.0026 (0.9677)	0.0046 (0.9989)	0.0084 (0.9982)	0.0017 (0.9945)	0.0020 (0.9971)	0.0014 (0.9869)	0.0017 (0.9945)	0.0019 (0.9951)	0.0025 (0.9975)
	*t*_*1/2*_ (day)	577.6	407.7	266.6	150.7	82.5	407.7	346.6	495.1	407.7	364.8	277.3
	*Ea* (kJ/mol)	21.59 (0.9823)										
Acteoside Standard	*k* (day^−1^)	0.0015 (0.9697)	0.0054 (0.9833)	0.0246 (0.9944)	0.4269 (0.9928)	1.9496 (0.9825)	0.0054 (0.9833)	0.0124 (0.9889)	0.004 (0.9945)	0.0054 (0.9833)	0.0135 (0.9830)	1.0451 (0.9801)
	*t*_*1/2*_ (day)	462.1	128.4	28.2	1.6	0.4	128.4	55.9	173.3	128.4	51.3	0.7
	*Ea* (kJ/mol)	81.83 (0.9576)										

*k*, the kinetics constant; *t*_*1/2*_, the half-life time; *Ea*, activation energy; PhGs, phenylethanoid glycosides; TPG, total phenylethanoid glycoside; OFE, *O. fragrans* var. *thunbergii* flower extracts.

The *k* values and *t*_*1/2*_ values of TPG (including 6.36% salidroside and 65.35% acteoside) in OFE ranged from 2.1 × 10^−3^ to 251 × 10^−3^ day^−1^ and 2.8 to 330.1 day (Table 1), respectively. At lower temperatures (4 °C and 20 °C) and lower pH values (pH 5.0 and pH 6.0), the *t*_*1/2*_ values of TPG were higher, while they decreased drastically at high temperatures (50 °C and 80 °C) and high pH values (pH 9.0). The *t*_*1/2*_ values of salidroside in OFE ranged from 20.8 to 177.7 day. At the same condition, the *t*_*1/2*_ value of acteoside in OFE was lower than that of salidroside, indicating that acteoside was less stable than salidroside. It was attributable to the different molecular structures of salidroside and acteoside. Salidroside is phenylethanoid monosaccharides, while acteoside is phenylethanoid disaccharide and has ester linkage, which is easy to be hydrolyzed. The calculated activation energy (*Ea*) values of TPG, salidroside and acteoside in OFE were 50.40, 21.63 and 69.14 kJ/mol, respectively (Table 1). Higher *Ea* values indicate stronger temperature dependence, that is the reaction running slowly at low temperature but fast at high temperature^27^. So acteoside in OFE was more susceptible to temperature elevation during heating.

The *t*_*1/2*_ values of salidroside and acteoside standards were higher than those of salidroside and acteoside in OFE with same treatments. The results showed that the standards of salidroside and acteoside were more stable than those in OFE under the same storage condition. As the OFE was crude extract (including enzyme, organic acids, etc.), some of these compounds might accelerate the degradation^28^ of salidroside and acteoside in OFE, compared with the standards.

These findings revealed that the degradation of PhGs follows first-order reaction kinetic, which could be used to predict their contents during storage. Phenylethanoid glycosides were unstable at high temperature, high pH and light exposure conditions, therefore PhGs should be stored in the dark at low temperature and pH.

### Main degradation products of salidroside and acteoside

**Figure 2:**
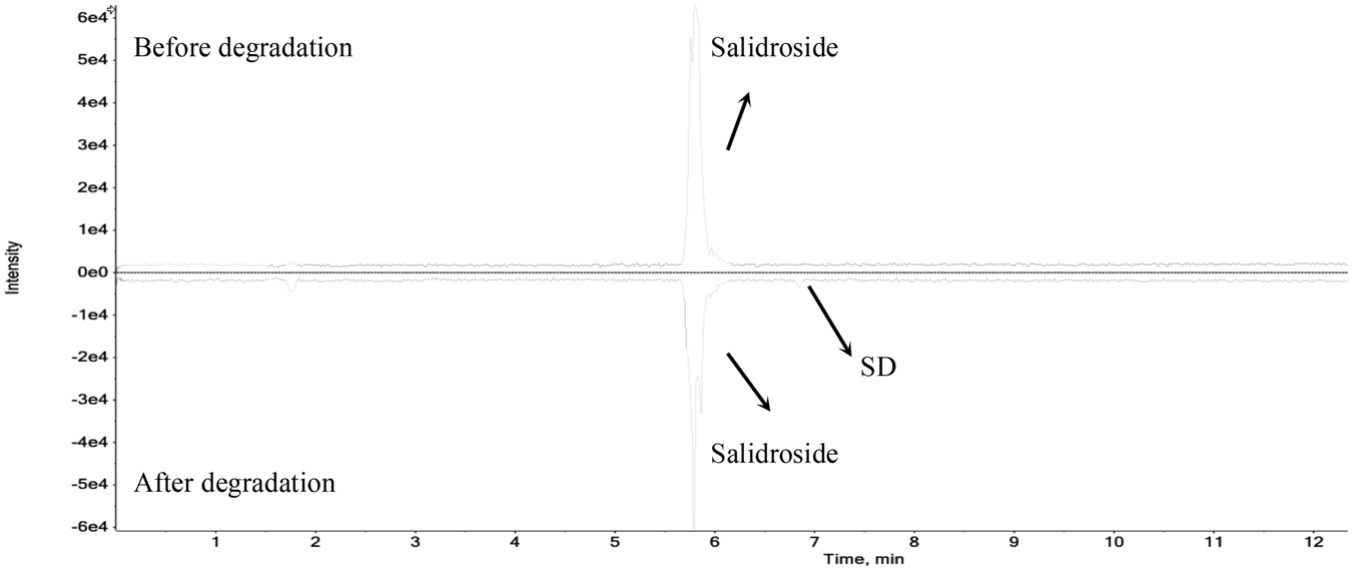
Total ion chromatogram of salidroside before and after degradation in positive ion mode. SD: salidroside degradation product.

**Figure 3:**
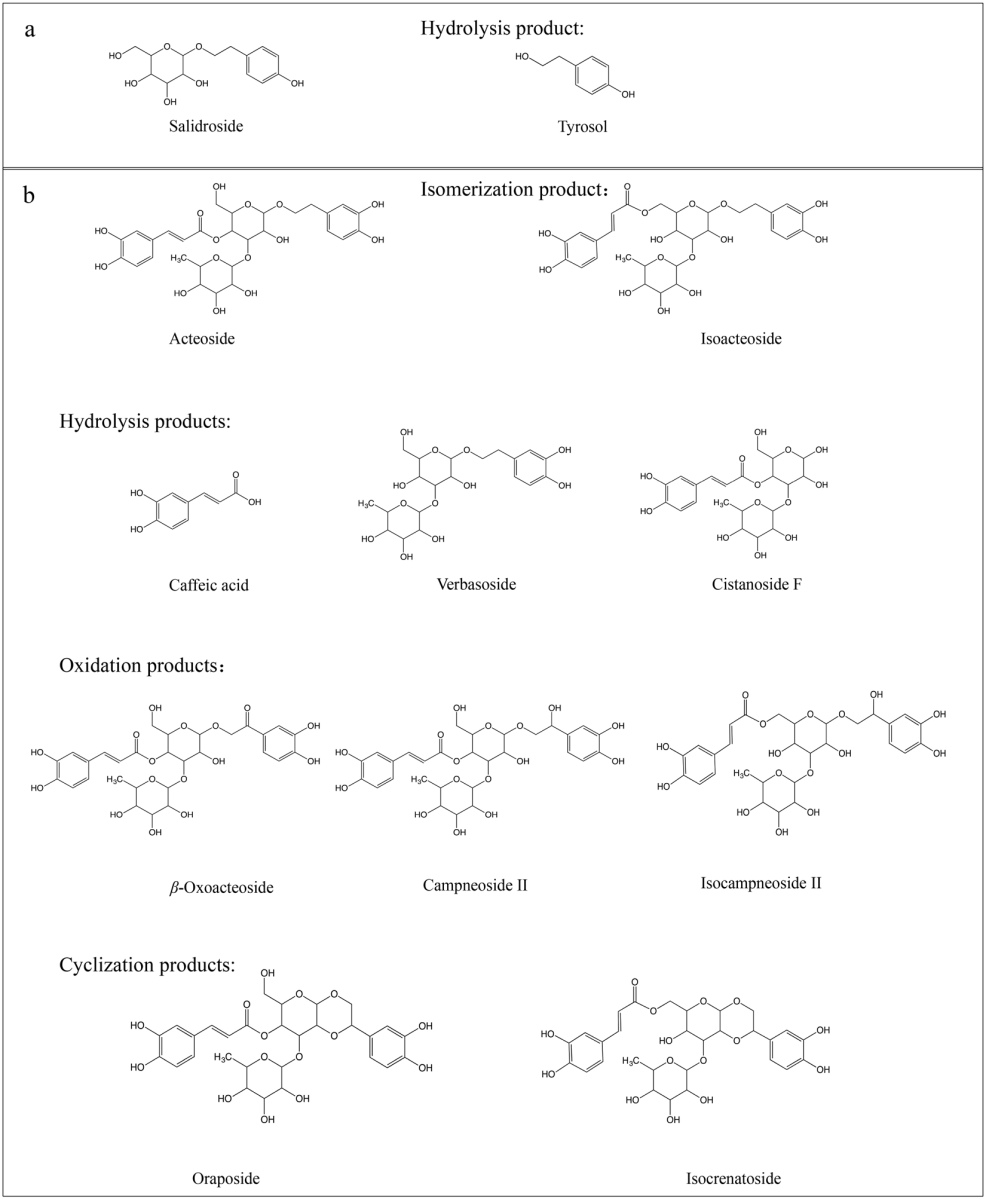
Chemical structures of salidroside (**a**), acteoside (**b**) and their possible degradation products.

For tentative identification of the degradation products, the degraded salidroside and acteoside were analyzed by UPLC–QTOF–MS/MS. Tyrosol, salidroside, caffeic acid, acteoside and isoacteoside were further identified using standards, and other degradation products were tentatively identified using the MS data. As shown in Fig. 2 and Supplementary Fig. S1, salidroside degradation product (SD) was the main degradation product of salidroside for all experiment treatment. Comparing the mass data with tyrosol standard, SD was identified as tyrosol, which was the aglycone of salidroside (structures shown in Fig. 3a). It indicated that salidroside was mainly hydrolyzed to tyrosol during storage. The MS/MS spectrum and proposed fragmentation pathway of salidroside and tyrosol are shown in Supplementary Fig. S2.

**Figure 4:**
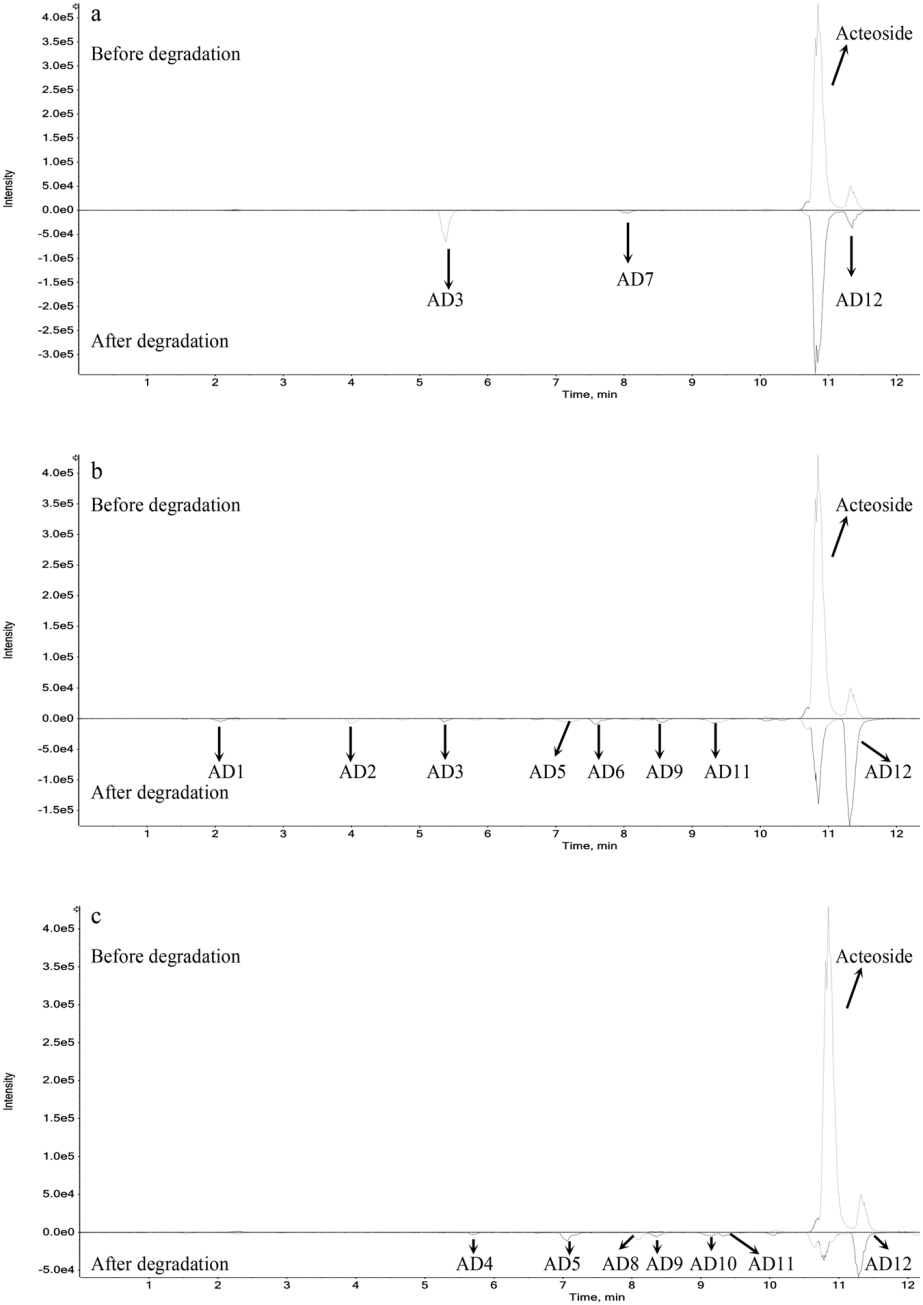
Total ion chromatograms of acteoside before and after degradation in negative ion mode in different storage conditions. (**a**) In common storage conditions (temperature ≤37 °C, pH ≤ 7); (**b**) at high temperature; (**c**) at high pH. AD: acteoside degradation product.

Acteoside generated more degradation products (Supplementary Fig. S3) than salidroside during storage. As shown in Fig. 3b, acteoside was composed of four chemical moieties, including caffeic acid, hydroxytyrosol (phenylethanoid aglycone), glucose (central saccharide) and rhamnose. The ester linkage, linking caffeic acid and glucose, could be easily hydrolyzed under some conditions. At the lower temperatures (≤37 °C), acteoside was hydrolyzed to verbasoside and caffeic acid (Fig. 4a, Table 2 and Supplementary Fig. S3a), and also isomerized into isoacteoside, while at high temperatures (50 and 80 °C, Fig. 4b), acteoside were hydrolyzed to verbasoside and isomerized into isoacteoside. Acteoside and isoacteoside were further oxidized to campneoside II and isocampneoside II, respectively. Acteoside was also oxidized to *β*-oxoacteoside, and the degradation products of AD1, AD5 and AD6 were identified as unknown compounds (Table 2) according to the current information.

**Table 2:** Retention time, mass measurements, and predicted formulas of acteoside and its degradation products.

Degradation product	Retention time (min)	Precursor ion [M-H]^−^ m/z	Fragment ion [M-H]^−^ m/z	Error (ppm)	Formula	Identification
AD1	2.092	383.1178	237.0607, 193.0704, 129.0195, 75.0099	−4.4	C_14_H_24_O_12_	Unkonwn
AD2	4.008	637.1740	619.1691, 491.1166, 311.0564, 179.0351	−1.6	C_29_H_34_O_16_	*β*-Oxoacteoside
AD3	5.362	461.1647	315.1080, 161.0452, 135.0451	−3.1	C_20_H_30_O_12_	Verbasoside
AD4	5.819	487.1432	179.0339, 179.0339, 135.0444	−4.2	C_21_H_28_O_13_	Cistanoside F
AD5	7.125	635.1586	399.0709, 309.0377, 283.0594, 265.0491	−1.5	C_29_H_32_O_16_	Unkonwn
AD6	7.584	619.1688	383.0741, 311.0551, 267.0646, 241.0483	−1.5	C_29_H_32_O_15_	Unkonwn
AD7	8.037	179.0354	161.0253, 135.0448	2.3	C_9_H_8_O_4_	Caffeic acid
AD8	8.11	621.1786	475.1216, 179.0341, 135.0491	−3.9	C_29_H_34_O_15_	Oraposide
AD9	8.426	639.1926	621.1812, 487.1444, 179.0332, 161.0234, 151.0378, 135.0474	−1.8	C_29_H_36_O_16_	Campneoside II
AD10	9.127	621.1783	475.1235, 179.0351, 135.0449	−5	C_29_H_34_O_15_	Isocrenatoside
AD11	9.301	639.1934	621.1824, 487.1407, 459.1487, 179.0335, 151.0396	−1.2	C_29_H_36_O_16_	Isocampneoside II
Acteoside	10.85	623.1993	461.1667, 315.1087, 179.0351, 161.0251, 135.0454	−3.1	C_29_H_36_O_15_	Acteoside
AD12	11.27	623.1953	461.1648, 179.0339, 161.0237, 135.0445	−3.3	C_29_H_36_O_15_	Isoacteoside

AD, acteoside degradation products.

Exposure to light compared to the dark, it increased hydrolysis of acteoside to verbasoside and caffeic acid, and isomerization to isoacteoside (Fig. 4a, Table 2 and Supplementary Fig. S3b). In previous study, D’Imperio^25^ found that acteoside was quite unstable at pH 7 and could isomerize to isoacteoside. In the present study, when pH values ranged from 5.0 to 7.0 (all samples stored in the dark), we found the main degradation products of acteoside were verbasoside, caffeic acid and isoacteoside (Fig. 4a, Table 2 and Supplementary Fig. S3c), indicating that acteoside was mainly hydrolyzed and isomerized in acidic or neutral solution at 20 °C. However, in alkaline solution (pH 9.0), acteoside were hydrolyzed to cistanoside F and isomerized into isoacteoside (Fig. 4c, Table 2 and Supplementary Fig. S3c). Further, acteoside was oxidized to campneoside II and cyclized to oraposide, while isoacteoside was oxidized to isocampneoside II and cyclized to isocrenatoside. The degradation product of AD5 could not be tentatively identified according to the current information. The MS/MS spectrum and proposed fragmentation pathway of acteoside and its degradation products are shown in Supplementary Fig. S4.

At low temperature, acteoside isomerized into isoacteoside, and was hydrolyzed to verbasoside and caffeic acid in acidic or neutral solution, irrespective of storage in the dark or light. However, under high temperature and alkaline conditions, acteoside could also be cyclized to oraposide, and oxidized to campneoside II and *β*-oxoacteoside.

### Protective effect of phenylethanoid glycosides against CoCl_2_-induced death in PC12 cells

**Figure 5:**
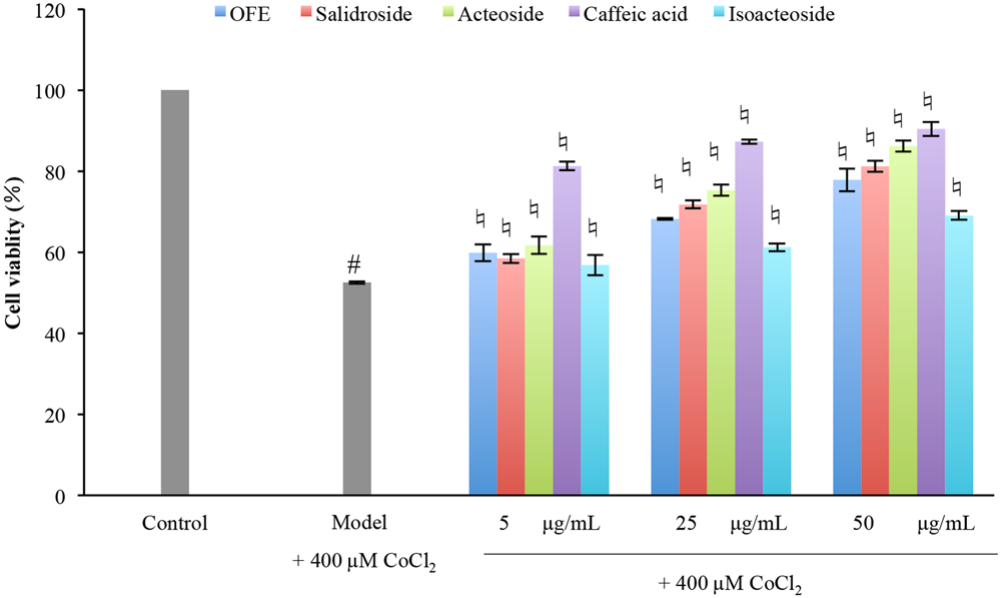
Protective effect of OFE, salidroside, acteoside, caffeic acid and isoacteoside on CoCl_2_-induced hypoxia damage in PC-12 cell. OFE, *O. fragrans* var. *thunbergii* flower extracts; the 5, 25 and 50 μg/mg were the contents of total phenylethanoid glycoside in OFE. Cell viabilities were measured by CCK-8 assay. ^#^ *p* < 0.05 compared with control group; ^♮^ *p* < 0.05 compared with model group (+400 µM CoCl_2_).

The 400 μM CoCl_2_ treatment for 12 h induced significant decrease in cell viability (52.52 ± 0.31% viability) of cultured PC12 cells (Fig. 5), as compared with that of control cells (100% viability). As shown in Fig. 5, treatment with salidroside (5, 25 and 50 μg/mL), acteoside (5, 25 and 50 μg/mL) and OFE (TPG contents: 5, 25 and 50 μg/mL) significantly attenuated the CoCl_2_-induced decrease in cell viabilities in a concentration dependent way. The treatment with 50 μg/mL (167 μM) salidroside showed the maximum protection (81.15 ± 1.33% viability) among the given concentrations of salidroside, slightly higher than that of the treatment with 90 μM salidroside (79.6% viability) in a previous study^21^. Also, the treatment with 25 μg/mL (83 μM) salidroside showed lower cell viability (71.74 ± 0.93%) than that in the previous study. The cell viabilities of PC12 cells treated with 5, 25 and 50 μg/mL acteoside (equal to 8, 40 and 80 μM) were 61.67%, 75.24% and 86.13%, respectively, higher than those of salidroside (58.44%, 71.74% and 81.15% for 17, 83 and 167 μM, respectively). It revealed that acteoside could protect PC12 cells against CoCl_2_-induced hypoxia damage, even stronger than salidroside. In addition, the TPG, salidroside and acteoside contents showed good correlation with anti-hypoxia activities, with correlation coefficients of 0.945, 0.982 and 0.983 (*p* < 0.01), respectively.

**Figure 6:**
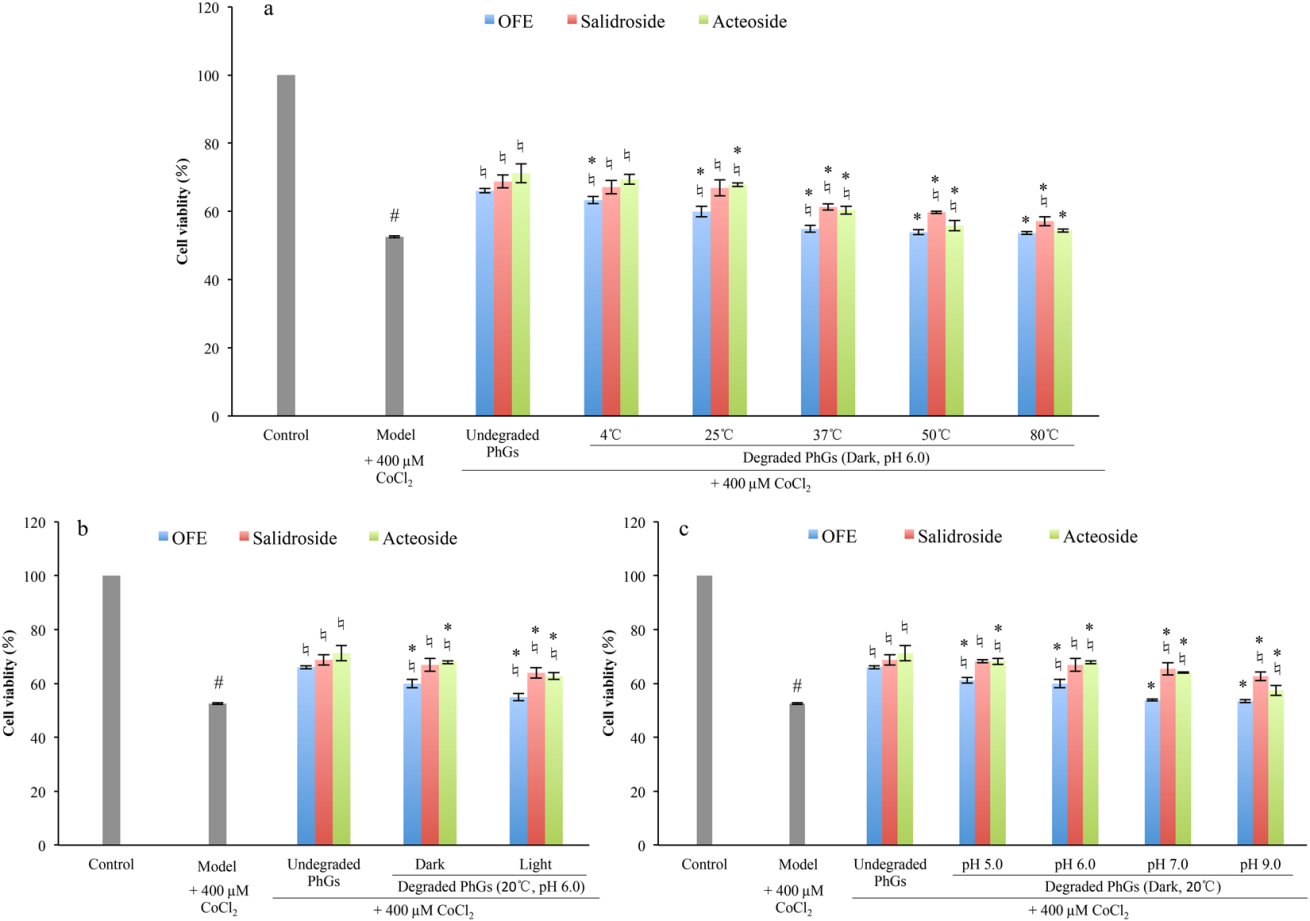
Protective effects of degraded PhGs on CoCl_2_-induced hypoxia damage in PC12 cell. a, cell viabilities of PC12 cells treated with PhGs stored at different temperature at pH 6.0 in the dark; b, cell viabilities of PC12 cells treated with PhGs stored at different light exposure at pH 6.0 at 20 °C; c, cell viabilities of PC12 cells treated with PhGs stored at different pH at 20 °C in the dark. PhGs, phenylethanoid glycosides; OFE, *O. fragrans* var. *thunbergii* flower extracts. Cell viabilities were measured by CCK-8 assay. ^#^ *p* < 0.05 compared with control group; ^♮^ *p* < 0.05 compared with model group (+400 µM CoCl_2_); ^*^ *p* < 0.05 compared with undegraded PhGs in the same color.

As mentioned in the first part of Result and Discussion, the TPG, salidroside and acteoside contents decreased after degradation. Obviously, the cell viability of PC12 cells treated with degraded PhGs was lower compared with that of undegraded PhGs (Fig. 6). The cell viabilities of PC12 cells treated with OFE at high temperatures (Fig. 6a) and high pH values (Fig. 6c) were similar to that of model group, for acteoside and salidroside had almost disappeared in these treated samples. As shown in Fig. 6, treatment with degraded salidroside significantly attenuated the CoCl_2_-induced decrease in cell viabilities of PC12 cells, though the cell viabilities of PC12 cells treated with degraded salidroside were lower, compared with that of undegraded salidroside (Fig. 6).

For the degraded acteoside, the cell viabilities decreased (Fig. 6) as the acteoside concentrations declined. Of particular note, the concentrations of acteoside at light (pH 6.0, 25 °C, Fig. 1J) and pH 7.0 (25 °C, dark, Fig. 1O) were 33.13 μg/mL and 31.41 μg/mL, respectively. The final acteoside concentrations of these samples were approximately 3 μg/mL in the cell experiment. However, we found that the cell viabilities in the presence of degraded acteoside by light (62.88%, Fig. 6b) and pH 7.0 (64.00%, Fig. 6c) were higher than that of 5 μg/mL acteoside (61.67%). Under these storage conditions, the degradation products of acteoside were caffeic acid, isoacteoside and verbasoside. As shown in Fig. 5, the cell viability of PC12 cells treated with 5 μg/mL (equal to 28 μM) caffeic acid (81.22%) were higher than that of acteoside (75.24% for 40 μM), whereas the cell viabilities of PC12 cells treated with 5, 25 and 50 μg/mL (equal to 8, 40 and 80 μM) isoacteoside (56.81%, 61.19% and 68.99%, respectively) were significantly lower (*p* < 0.05) than those of acteoside. The caffeic acid and isoacteoside contents also showed good correlation with anti-hypoxia activities, with correlation coefficients of 0.94 and 0.96 (*p* < 0.01), respectively. We considered that caffeic acid might increase anti-hypoxia ability of degraded acteoside.

In conclusion, the degradation of PhGs (TPG, salidroside and acteoside) fitted to the first-order reaction kinetics, and temperature had the greatest effect on the *k* and *t*_*1/2*_ values, followed by pH. It suggested that PhGs should be stored at low temperature, low pH and dark condition. During storage, salidroside was mainly hydrolyzed to tyrosol, and acteoside was hydrolyzed to verbasoside and caffeic acid. Acteoside also could isomerize into isoacteoside, cyclize to oraposide, and oxidize to campneoside II, *β*-oxoacteoside, etc. The degradation attenuated the anti-hypoxia ability of PhGs, though caffeic acid could slightly increase the anti-hypoxia ability of degraded acteoside. It is necessary to use some methods to increase the stability of PhGs in the further study.

## Methods

### Standards and reagents

Caffeic acid (purity ≥98%), salidroside (purity ≥98%) and acteoside (purity ≥98%) were purchased from Aladdin (Shanghai, China). Tyrosol (purity ≥98%) and isoacteoside (purity ≥98%) were purchased from Yuanye Biotechnology Co. (Shanghai, China). CoCl_2_ was purchased from Sigma-Aldrich (St. Louis, MO, USA). HPLC-grade acetonitrile and guaranteed-grade formic acid were obtained from Merck (Shanghai, China). Other chemicals and reagents (analytical grade) were purchased from Sinopharm Chemical Regent Co. (Shanghai, China).

### Sample preparation

The dried *O. fragrans* var. *thunbergii* flowers (Guilin, Guangxi, China, 10 g) were extracted with 95% ethanol (100 mL) for 12 h at 20 °C using a method of Jiang *et al*.^12^ with modification. The mixture was filtered by vacuum pump (YuKang, Shanghai, China). The filtrate was evaporated (YaRong, Shanghai, China) at 40 °C under a vacuum to dryness, and dissolved with water to a final concentration of 1 mg flower extract/mL (OFE, test solution). Salidroside and acteoside were dissolved with water to a concentration of 100 μg/mL (test solution). The test solutions were used directly after preparation.

### Stability study

The influence of temperature on the stability was studied at 4 °C, 20 °C, 37 °C, 50 °C and 80 °C at pH value of 6.0. Each test solution was divided into 30 mL portions in sealed glass bottles and kept away from light at 4 °C (in refrigerator), at room temperature (20 °C) and in a water bath (37 °C, 50 °C and 80 °C), respectively (±2 °C). The total phenylethanoid glycoside (TPG) content was determined at 0, 7, 14, 21, 30, 45, 60 and 90 days except for those stored at 80 °C (determined at 0, 1, 2, 3, 4, 5, 6 and 7 days). The salidroside content was determined at 0, 7, 14, 21, 30, 45, 60 and 90 days. The acteoside content was determined at 0, 7, 14, 21, 30, 45, 60 and 90 days except for those stored at 50 °C (determined at 0, 1, 2, 3, 4, 5, 6 and 7 days) and 80 °C (determined at 0, 0.125, 0.25, 0.5, 0.75, 1 and 2 days).

The effect of pH on the stability was studied at different pHs (5.0, 6.0, 7.0 and 9.0) at 20 °C. Hydrochloric acid (2 M) and sodium hydroxide (2 M) were used to adjust the pH. Each test solution was divided into 30 mL portions in sealed glass bottles, stored in the dark. The TPG and salidroside contents were determined at 0, 7, 14, 21, 30, 45, 60 and 90 days. The acteoside content was determined at 0, 7, 14, 21, 30, 45, 60 and 90 days except for pH value of 9.0 (determined at 0, 0.125, 0.25, 0.5, 0.75, 1 and 2 days).

A lamp (OSRAM DULUX S 11 W/865, Hangzhou, China) was used in the light exposure experiment, performed at 20 °C. Each test solution (pH value of 6.0) was divided into 30 mL portions in sealed glass bottles and placed 20 cm under the lamp with a light intensity of 2000 Lux, which was detected by a light meter (Victor 1010 A, Shenzhen, China). The TPG, salidroside and acteoside contents were determined at 0, 7, 14, 21, 30, 45, 60 and 90 days.

The treatment was performed in triplicates (n = 3). Samples before storage and collected after completion of storage at above conditions were subjected to MS analysis.

### Total phenylethanoid glycoside content determination

The total phenylethanoid glycoside content was determined using a method described by Jiang *et al*.^12^. A Biotek microplate reader (Winooski, VT, USA) was used to measure the absorbance of the OFE at 334 nm. The contents were expressed as micrograms of acteoside equivalents (AE) per milliliters in OFE solution (μg AE/mL). The concentration range of the calibration series was 5 to 200 μg/mL.

### UHPLC–DAD analysis

The samples were filtered through a 0.22 μm nylon syringe filter (ANPEL, Shanghai, China) and analyzed by an Agilent 1290 UHPLC instrument (Agilent, Waldbronn, Germany) equipped with an autosampler, a binary pump, a column thermostat and a diode-array detector, using a previous method^13^ with modification. The sample was separated on an Agilent ZORBAX Eclipse XDB-C18 column (3.5 μm, 2.1 mm × 150 mm) at 25 °C. The mobile phase consisted of acetonitrile (solvent A) and water (containing 0.1% formic acid, solvent B). A gradient program was used according to the following profile: 0–1 min, 6% A; 1–4 min, from 6% to 15% A; 4–8 min, from 15% to 20% A; 8–10 min, from 20% to 30% A; 10–12 min, from 30% to 100% A; 12–12.5 min, from 100% to 6% A; 12.5–15 min, 6% A. The flow rate was 0.2 mL/min and the injection volume was 2 μL. The DAD detector was set from 190 nm to 400 nm.

### UPLC–QTOF–MS/MS analysis

The samples were filtered through a 0.22 μm nylon syringe filter (ANPEL, Shanghai, China) and transferred to an autosampler vial for UPLC–PDA–QTOF–MS analysis. The UPLC analyses were performed on a Waters ACQUITY UPLC (Waters, Milford, MA, USA) equipped with an autosampler, a binary pump, a column thermostat and a photo-diode array, using a previous method^13^ with modification. An Agilent ZORBAX Eclipse XDB-C18 column (3.5 μm, 2.1 mm × 150 mm) was used at 25 °C. The mobile phase and gradient program were described as the same as the UHPLC-DAD analysis. The flow rate was 0.2 mL/min and the injection volume was 2 μL. The PDA detector was set 280 nm.

The UPLC system coupled with a Triple TOF 5600^+^ mass spectrometer (AB SCIEX, Framingham, USA). In the negative ion mode, the source voltage was −4.5 kV and the source temperature was 550 °C; in the positive ion mode, the source voltage was 5.5 kV and the source temperature was 600 °C. The other MS conditions were set as follows: scan range, m/z 50–1500; nebulizer gas (Air), 50 psi; heater gas (Air), 50 psi; curtain gas (N_2_), 35 psi; maximum allowed error, ± 5 ppm; declustering potential (DP), 100 V; collision energy (CE), 10 V. For MS/MS acquisition mode, the parameters were the same except that the collision energy (CE) was set at 40 ± 20 V, ion release delay (IRD) at 67, ion release width (IRW) at 25. In addition, information-dependent acquisition (IDA)-based auto-MS^2^ was performed on the 8 most intense metabolite ions in a cycle of full scan (1 s). The exact mass calibration was performed automatically by the Automated Calibration Delivery System before each analysis.

MS data were acquired using an Analyst® TF 1.6 software (AB Sciex) and processed by PeakView 1.2 (AB Sciex). The degradation products of acteoside and salidroside were tentatively identified based on the reported literatures and free accessible databases, such as Reaxys^29^, ChemSpider^30^, Metlin Metabolite^31^ and MassBank^32^.

### Cell culture

The differentiated rat pheochromocytoma cell line PC12 was obtained from the Cell bank of Chinese Academy of Sciences (Shanghai, China) and cultured in RPMI-1640 medium (Hyclone, Logan, UT, USA), supplemented with 100 U/mL penicillin, 100 μg/mL streptomycin and 10% fetal bovine serum (Hyclone, Logan, UT, USA) in a humidified incubator with 5% CO_2_ at 37 °C^33^.

### Cell viability assay by CCK-8 assay

Cell viability was determined by Cell Counting Kit-8 (CCK-8)^34^ (Jiancheng, Nanjing, China). PC12 cells were placed in 96-well plates at a density of 1 × 10^4^ cells per well and in a volume of l80 μL. The plates were cultured for 24 h, and 20 μL of samples were added to the experimental groups. The control and model groups were added 20 μL of culture medium. After 24 h of incubation, the culture medium was replaced with 100 μL medium containing 400 µM CoCl_2_ while the control group was replaced with 100 μL new medium^21^. After 12 h treatment, 10 µL CCK-8 solution was added to each well and the plates were incubated at 37 °C for an additional 2 h. The absorbance was measured at 450 nm using Biotek microplate reader (Winooski, VT, USA) and the background absorbance was excluded by performing blank corrections. Cell viability was expressed as a percentage of the untreated group (control = 100%).

### Degradation kinetic analysis

The degradation kinetics of most biological substances in food system follow the zero-order equation (1), first-order equation (2) and second-order equations (3) reactions^35^.

1 $${\rm{Zero}}-{\rm{order}}:C-{C}_{0}=-kt\,$$

2 $${\rm{First}}-{\rm{order}}:lnC-ln{C}_{0}=-kt$$

3 $${\rm{Second}}-{\rm{order}}:\frac{1}{C}-\frac{1}{{C}_{0}}=-kt$$

where *C*_0_ and *C* are the PhGs contents (μg/mL) at time *t*_0_ and *t*, respectively; *k* is the rate constant (day^−1^); and *t* is the storage time (day).

The degradation of PhGs fitted to the first-order kinetic equation and the half-live time^36^ (*t*_*1/2*_, the time needed for 50% degradation of PhGs) were calculated by the following equations (4):

4 $${t}_{1/2}=-\frac{ln0.5}{k}$$

The temperature-dependence of the rate constant (*k*) can be expressed by the Arrhenius equation^27^ (5):

5 $$lnk=ln{K}_{0}-{E}_{a}/RT$$

where *K*_0_ is frequency factor (day^−1^); *Ea* is the Arrhenius activation energy (kJ/mol); *R* is the universal gas constant (8.314 J/(mol∙K)); and *T* is absolute temperature (in Kelvin, K). The *Ea* was calculated according to equation (5).

### Statistical analysis

All the analyses were performed in triplicate, and values were reported as the mean ± standard deviation. All results were confirmed from three independent experiments in the cell assay. Statistical analysis was performed using SPSS 20.0 and Excel 2011. One-way analysis of variance (ANOVA) were used to evaluate the significant differences between means, and *p* < 0.05 was considered to indicate statistical significance. Mathematical models were selected by comparing correlations coefficients and the 1stOpt Inst. software, version 15 pro (7D-Soft High Technology Inc., China) was used to calculate the parameters of kinetic models.

## Electronic supplementary material

Supplementary Information
