# Supplementary material for: Degradation of phenylethanoid glycosides in Osmanthus fragrans Lour. flowers and its effect on anti-hypoxia activity
Source: Sci Rep. 2017 Aug 30;7:10068. doi: 10.1038/s41598-017-10411-0 (PMC5577317; doi:10.1038/s41598-017-10411-0)
Supplement: Supplementary file 1 — Supplementary Information [file 41598_2017_10411_MOESM1_ESM.pdf]

Degradation of phenylethanoid glycosides in *Osmanthus fragrans* Lour. flowers and its effect on anti-hypoxia activity

Fei Zhou[1], Yajing Zhao[1], Maiquan Li[1], Tao Xu[1], Liuquan Zhang[1], Baiyi Lu[1]*, Xiaodan Wu[2], Zhiwei Ge[2]

[1]National Engineering Laboratory of Intelligent Food Technology and Equipment, Key Laboratory for Agro-Products Postharvest Handling of Ministry of Agriculture, Key Laboratory for Agro-Products Nutritional Evaluation of Ministry of Agriculture，Zhejiang Key Laboratory for Agro-Food Processing, Fuli Institute of Food Science, College of Biosystems Engineering and Food Science, Zhejiang University, Hangzhou, 310058, China

[2]Analysis Center of Agrobiology and Environmental Sciences, Zhejiang University, Hangzhou, 310058, China

*Corresponding author: Baiyi Lu, Email: bylu@zju.edu.cn, Tel./Fax: +86-0571-89882665

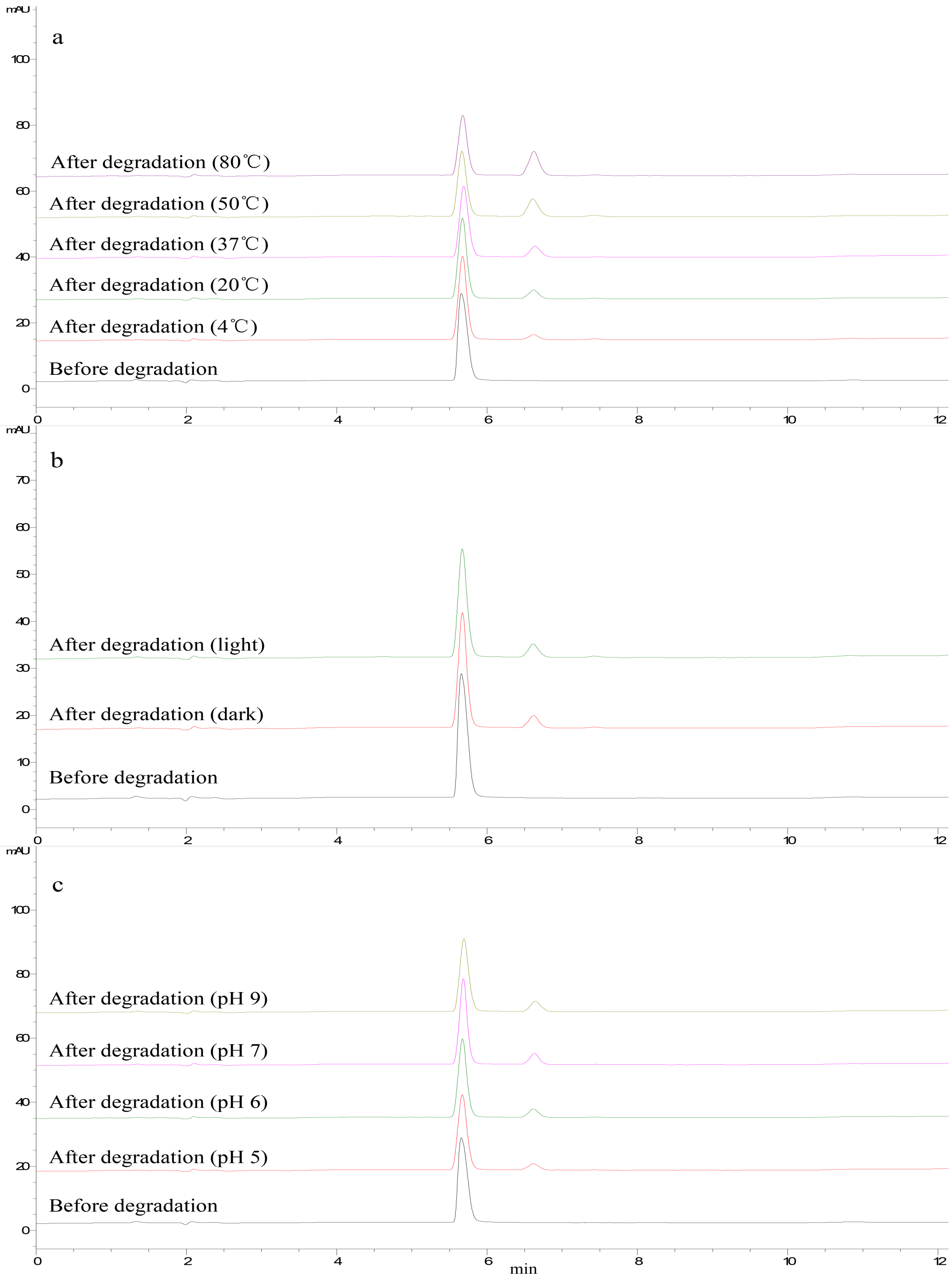

Supplementary Figure S1. UPLC–PDA chromatograms of salidroside before and after degradation under different conditions. a, UPLC–PDA chromatograms of salidroside before and after degradation at different temperature at pH 6.0 in the dark; b, UPLC–PDA chromatograms of salidroside before and after degradation at different light exposure at pH 6.0 at 20℃; c, UPLC–PDA chromatograms of salidroside before and after degradation at different pH at 20℃ in the dark.

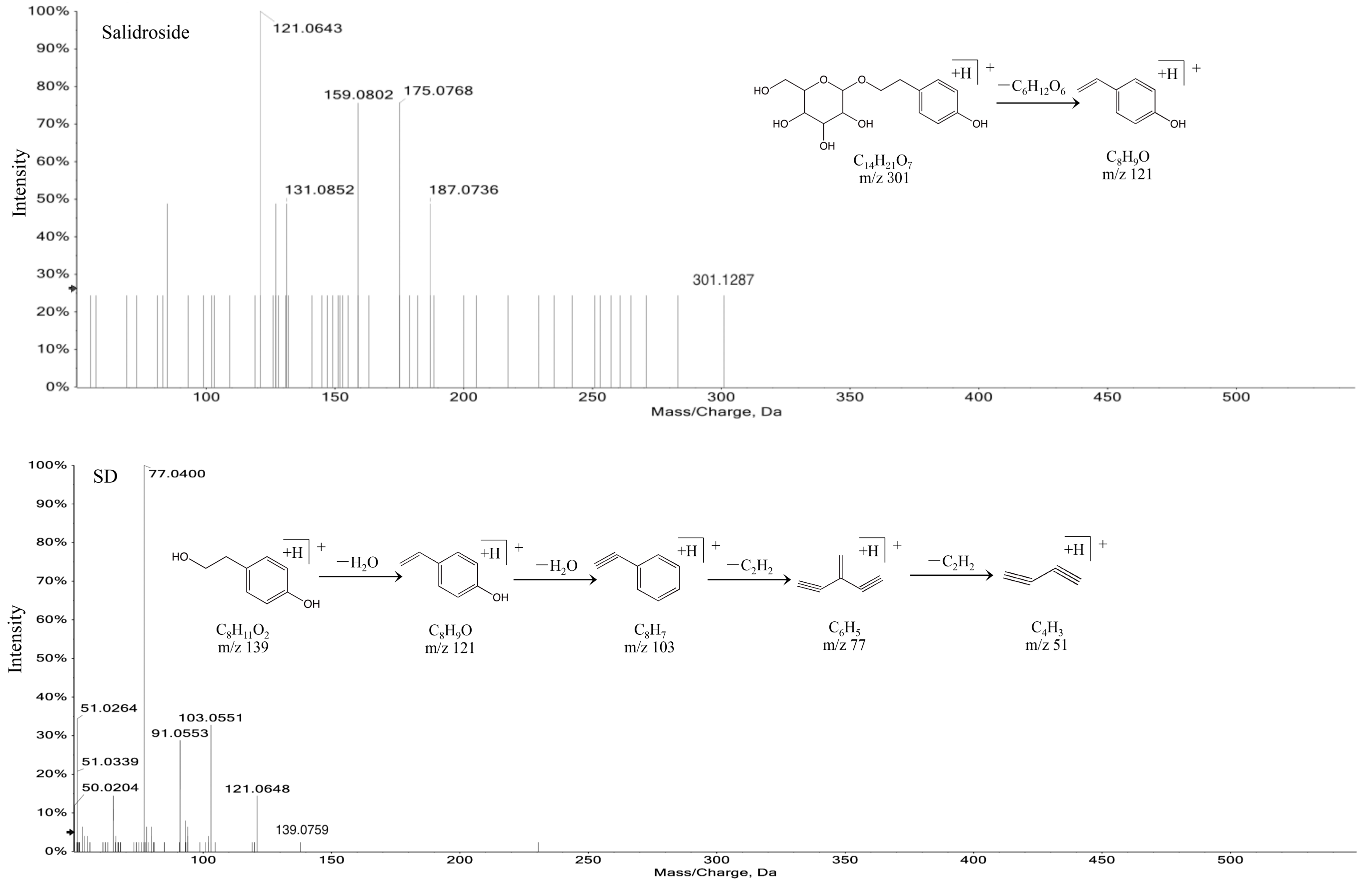

Supplementary Figure S2. MS/MS spectrum and proposed fragmentation pathway of salidroside and its degradation product (positive ionization mode). SD, salidroside degradation product (tyrosol).

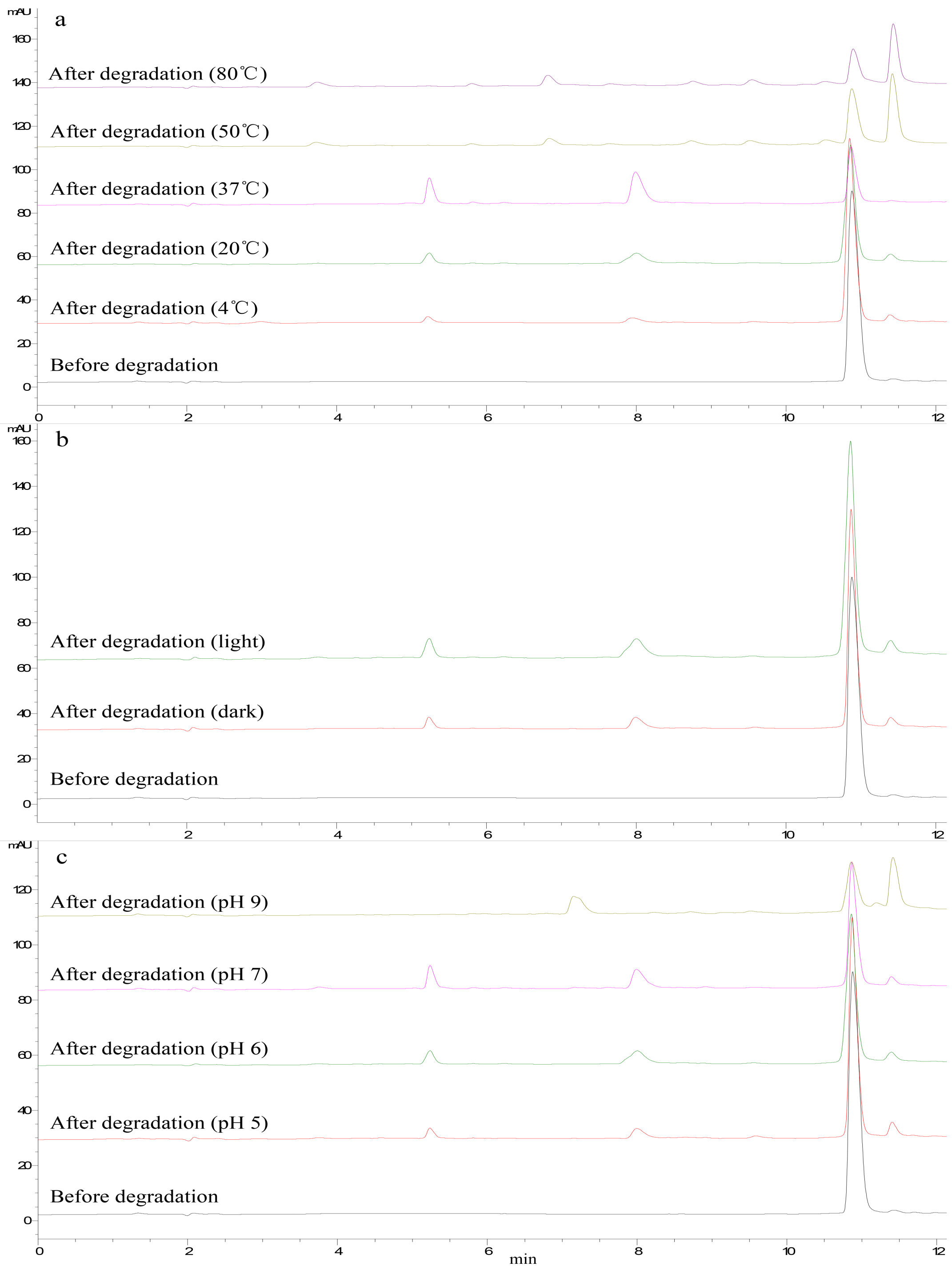

Supplementary Figure S3. UPLC–PDA chromatograms of acteoside before and after degradation under different conditions. a, UPLC–PDA chromatograms of acteoside before and after degradation at different temperature at pH 6.0 in the dark; b, UPLC–PDA chromatograms of acteoside before and after degradation at different light exposure at pH 6.0 at 20℃; c, UPLC–PDA chromatograms of acteoside before and after degradation at different pH at 20℃ in the dark.

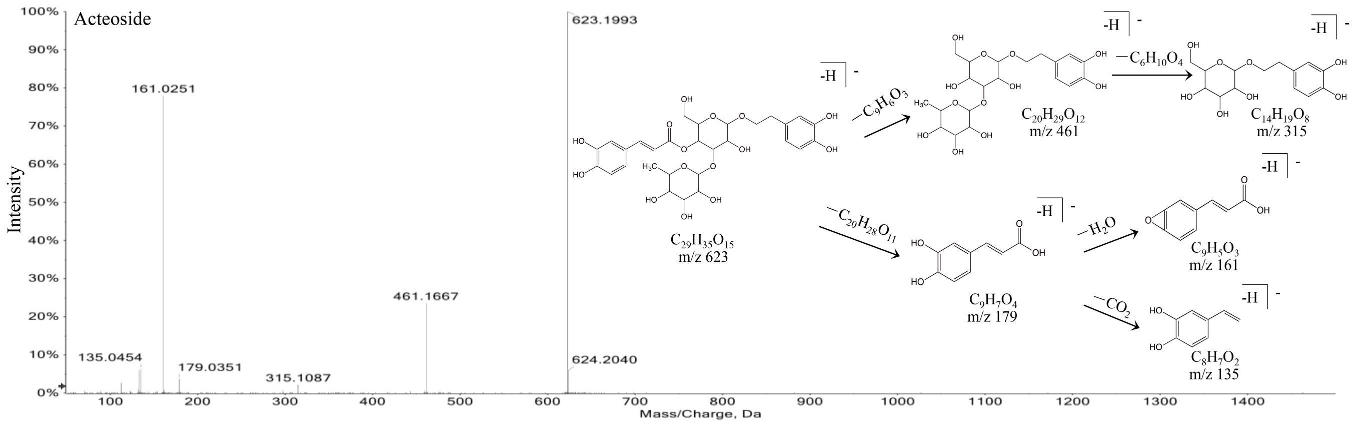
Acteoside
Intensity
Mass/Charge, Da
623.1993
161.0251
461.1667
135.0454
179.0351
315.1087
624.2040
$C_{29}H_{35}O_{15}$
m/z 623
$-C_9H_6O_3$
$C_{20}H_{29}O_{12}$
m/z 461
$-C_6H_{10}O_4$
$C_{14}H_{19}O_8$
m/z 315
$-C_{20}H_{28}O_{11}$
$C_9H_7O_4$
m/z 179
$-H_2O$
$C_9H_5O_3$
m/z 161
$-CO_2$
$C_8H_7O_2$
m/z 135

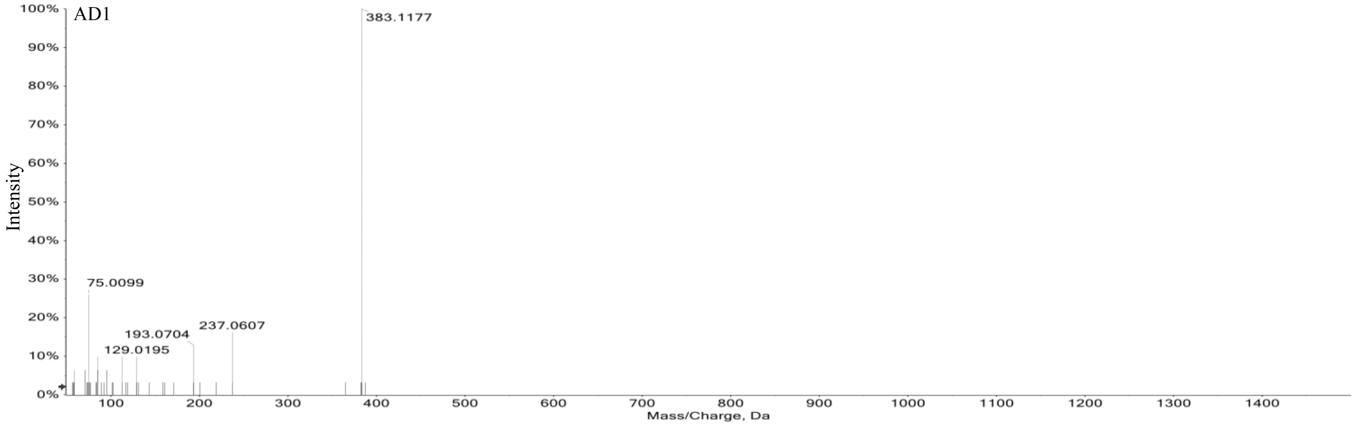
AD1
Intensity
Mass/Charge, Da
383.1177
75.0099
193.0704
129.0195
237.0607

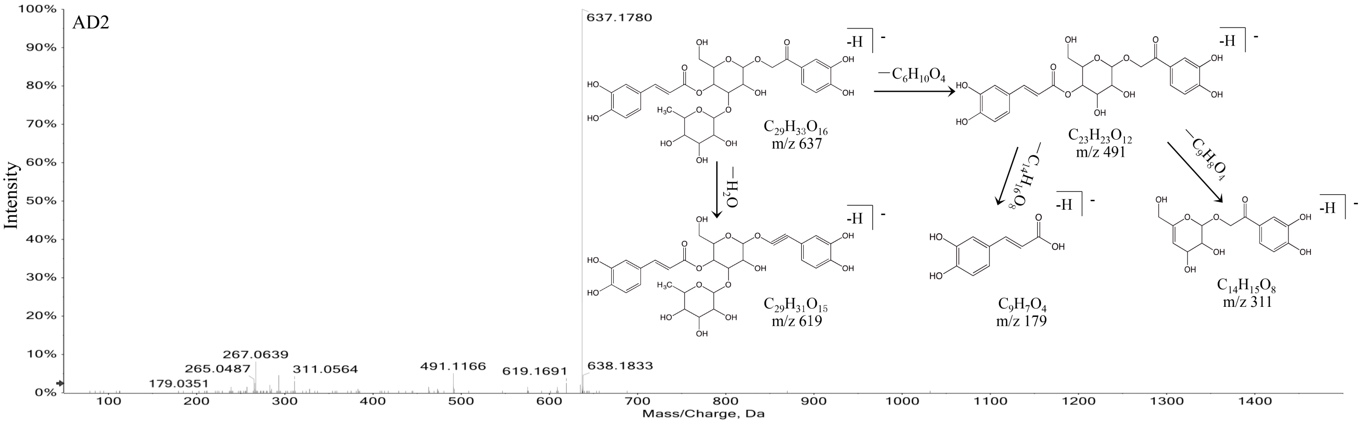
AD2
Intensity
Mass/Charge, Da
637.1780
267.0639
265.0487
179.0351
311.0564
491.1166
619.1691
638.1833
$C_{29}H_{33}O_{16}$
m/z 637
$-C_6H_{10}O_4$
$C_{23}H_{23}O_{12}$
m/z 491
$-H_2O$
$C_{29}H_{31}O_{15}$
m/z 619
$-C_{14}H_{16}O_8$
$C_9H_7O_4$
m/z 179
$-C_9H_8O_4$
$C_{14}H_{15}O_8$
m/z 311

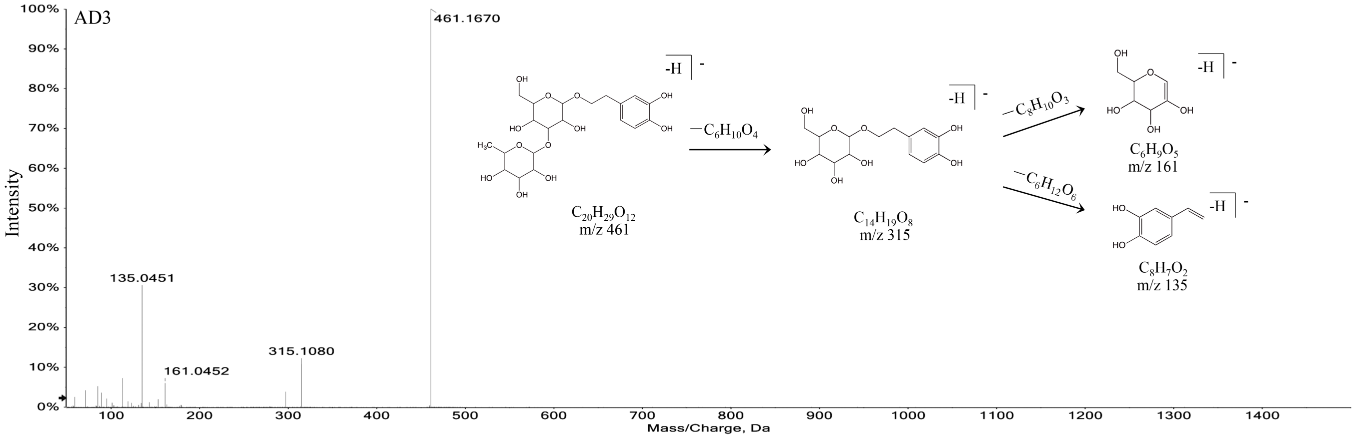
AD3
Intensity
Mass/Charge, Da
461.1670
135.0451
315.1080
161.0452
$C_{20}H_{29}O_{12}$
m/z 461
$-C_6H_{10}O_4$
$C_{14}H_{19}O_8$
m/z 315
$-C_8H_{10}O_3$
$C_6H_9O_5$
m/z 161
$-C_6H_{12}O_6$
$C_8H_7O_2$
m/z 135

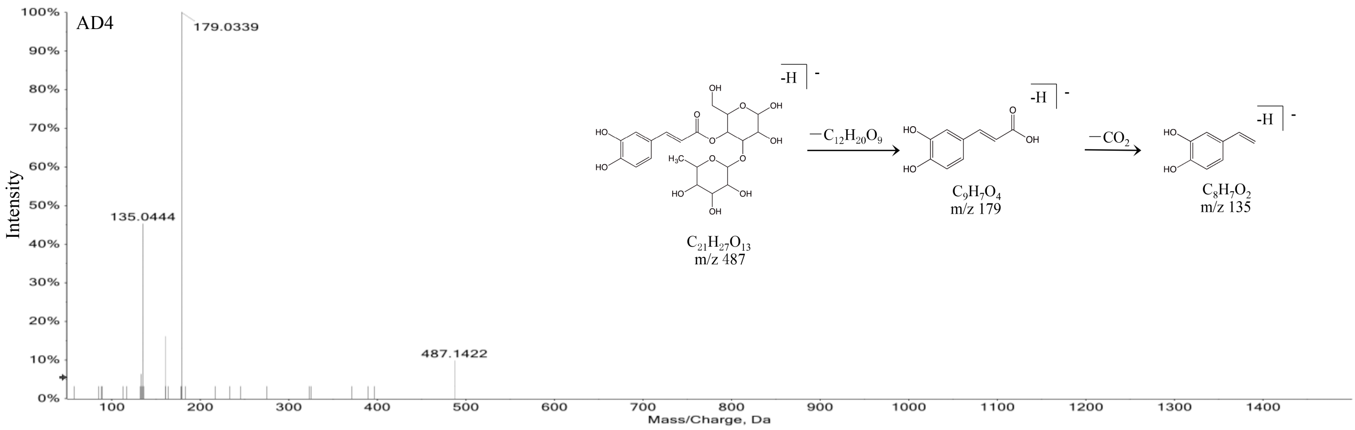
AD4
179.0339
135.0444
487.1422
Intensity
Mass/Charge, Da
−C12H20O9
−CO2
C21H27O13
m/z 487
C9H7O4
m/z 179
C8H7O2
m/z 135
-H -

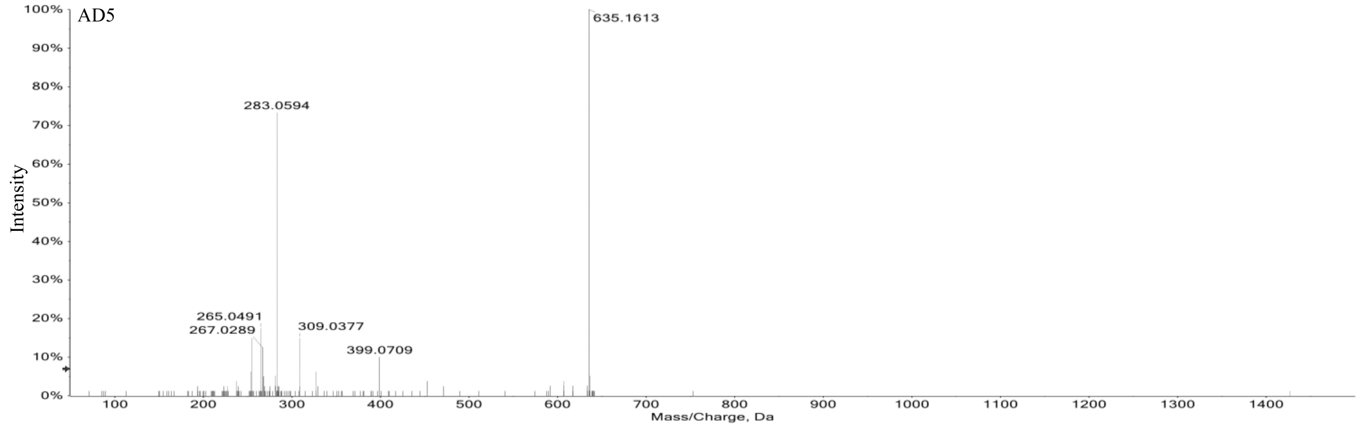
AD5
635.1613
283.0594
265.0491
267.0289
309.0377
399.0709
Intensity
Mass/Charge, Da

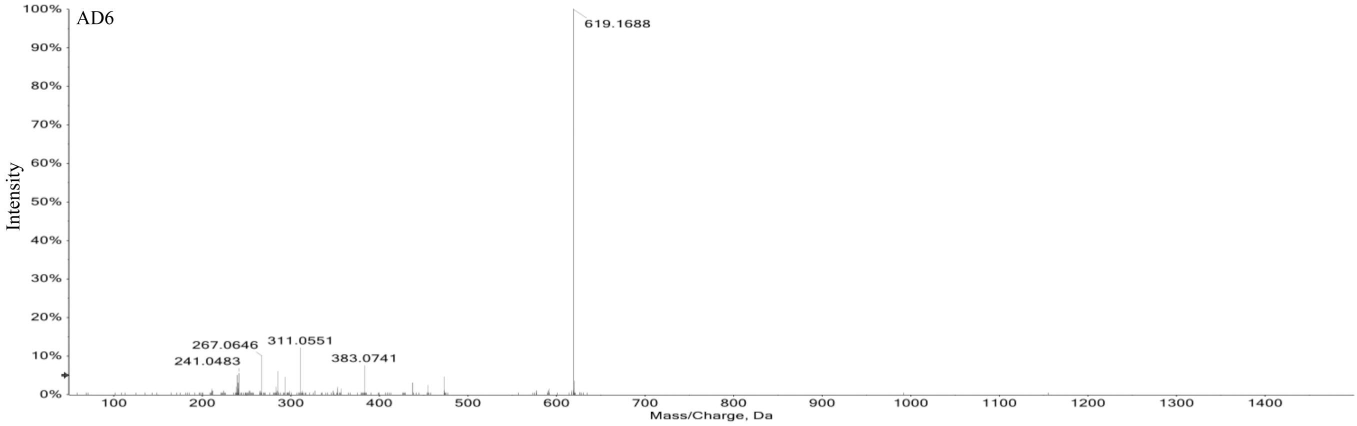
AD6
619.1688
267.0646
241.0483
311.0551
383.0741
Intensity
Mass/Charge, Da

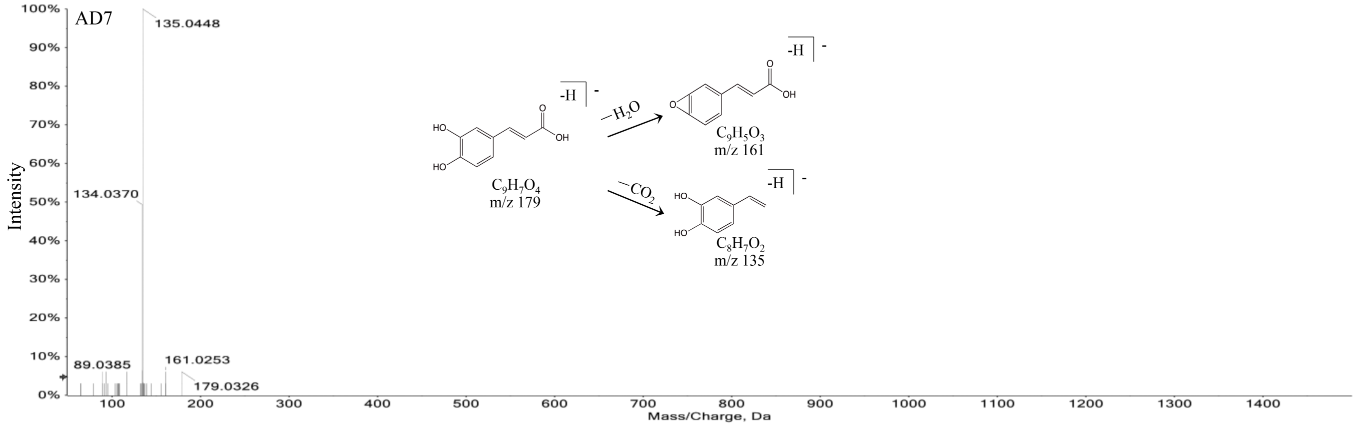
AD7
135.0448
134.0370
89.0385
161.0253
179.0326
Intensity
Mass/Charge, Da
−H2O
−CO2
C9H7O4
m/z 179
C9H5O3
m/z 161
C8H7O2
m/z 135
-H -

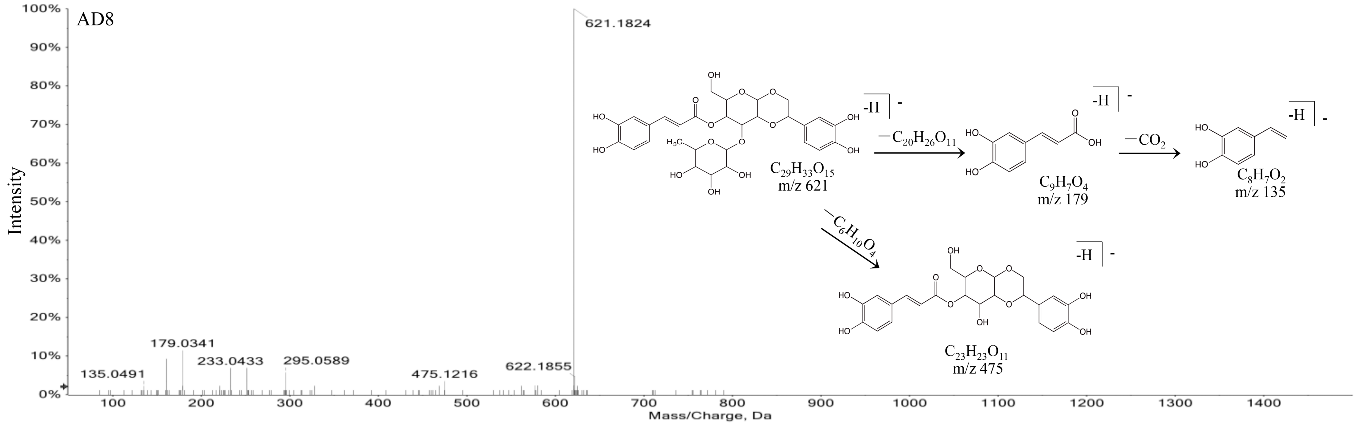
AD8
Intensity
Mass/Charge, Da
621.1824
179.0341
233.0433
295.0589
135.0491
475.1216
622.1855
-H
$-C_{20}H_{26}O_{11}$
$-CO_2$
$-C_6H_{10}O_4$
$C_{29}H_{33}O_{15}$
m/z 621
$C_9H_7O_4$
m/z 179
$C_8H_7O_2$
m/z 135
$C_{23}H_{23}O_{11}$
m/z 475

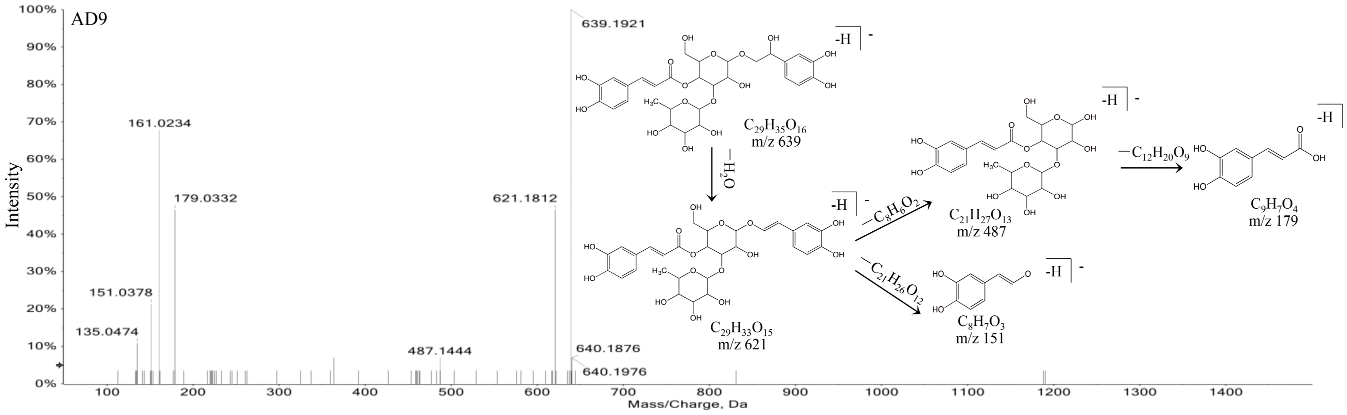
AD9
Intensity
Mass/Charge, Da
639.1921
161.0234
179.0332
621.1812
151.0378
135.0474
487.1444
640.1876
640.1976
-H
$-H_2O$
$-C_8H_6O_2$
$-C_{21}H_{26}O_{12}$
$-C_{12}H_{20}O_9$
$C_{29}H_{35}O_{16}$
m/z 639
$C_{29}H_{33}O_{15}$
m/z 621
$C_{21}H_{27}O_{13}$
m/z 487
$C_9H_7O_4$
m/z 179
$C_8H_7O_3$
m/z 151

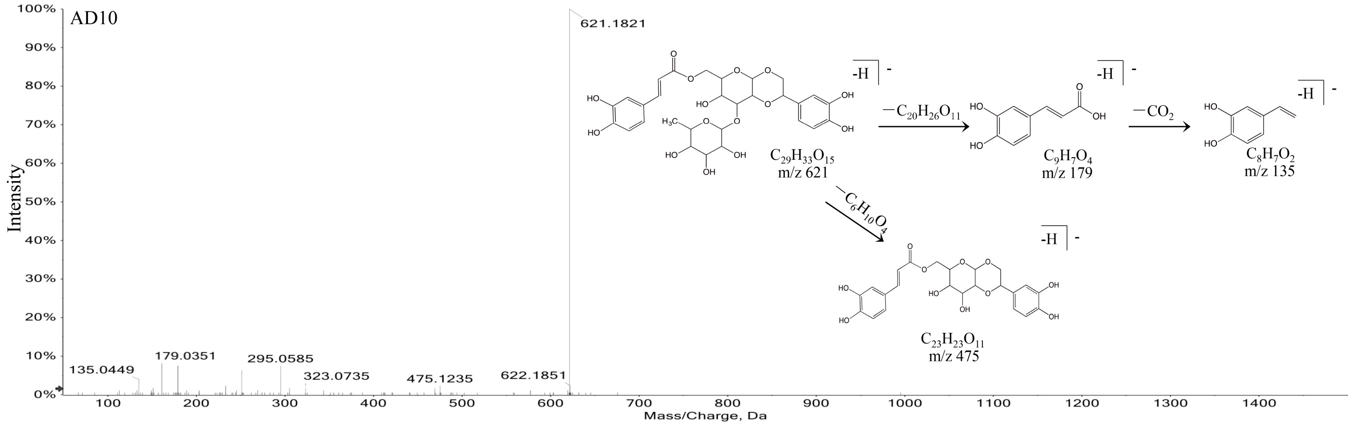
AD10
Intensity
Mass/Charge, Da
621.1821
135.0449
179.0351
295.0585
323.0735
475.1235
622.1851
-H
$-C_{20}H_{26}O_{11}$
$-CO_2$
$-C_6H_{10}O_4$
$C_{29}H_{33}O_{15}$
m/z 621
$C_9H_7O_4$
m/z 179
$C_8H_7O_2$
m/z 135
$C_{23}H_{23}O_{11}$
m/z 475

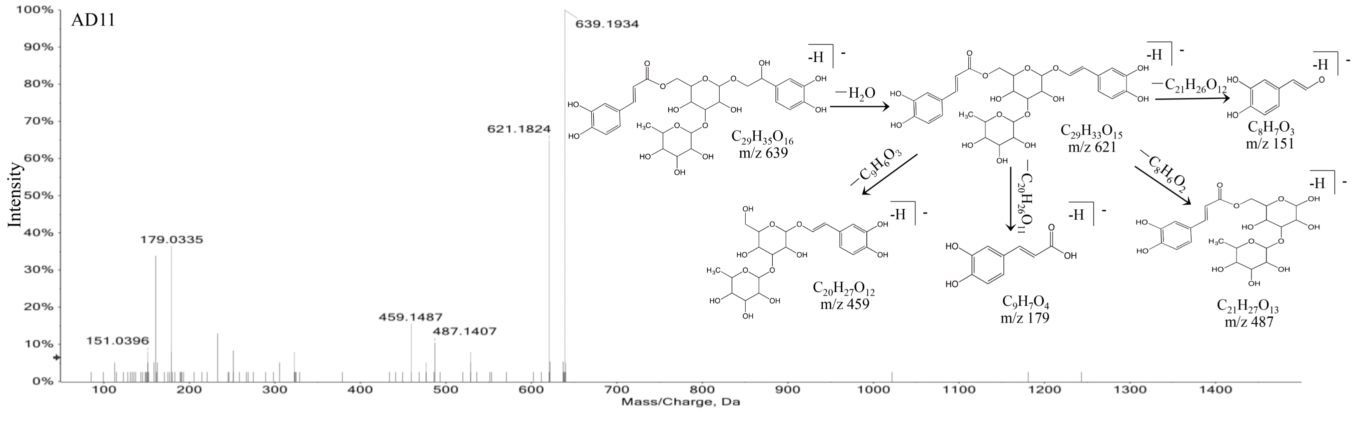
AD11
Intensity
Mass/Charge, Da
639.1934
621.1824
179.0335
459.1487
487.1407
151.0396
-H
$-H_2O$
$-C_{21}H_{26}O_{12}$
$-C_9H_6O_3$
$-C_{20}H_{26}O_{11}$
$-C_8H_6O_2$
$C_{29}H_{35}O_{16}$
m/z 639
$C_{29}H_{33}O_{15}$
m/z 621
$C_8H_7O_3$
m/z 151
$C_{20}H_{27}O_{12}$
m/z 459
$C_9H_7O_4$
m/z 179
$C_{21}H_{27}O_{13}$
m/z 487

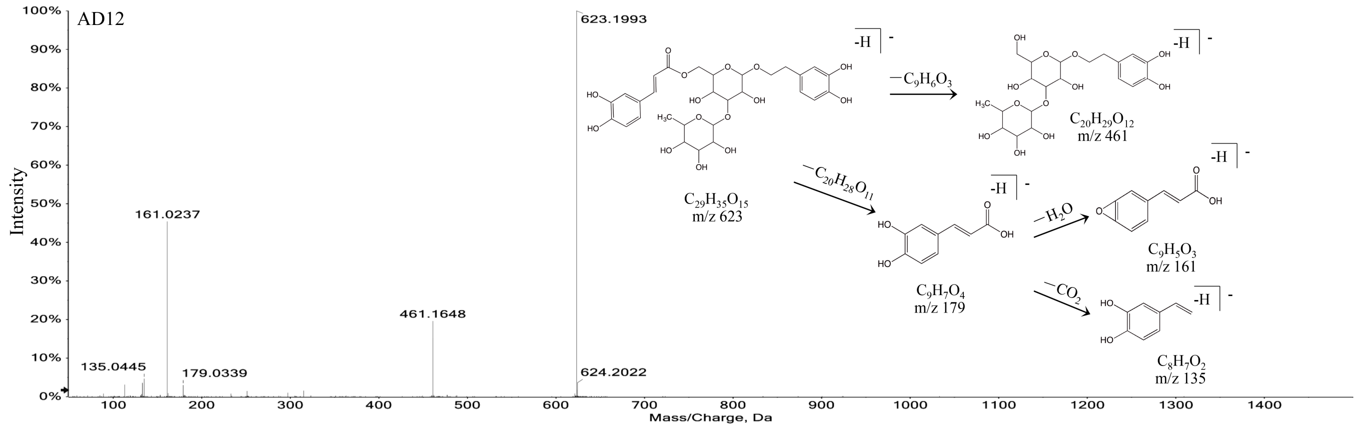

Supplementary Figure S4. MS/MS spectrum and proposed fragmentation pathway of acteoside and its degradation products (negative ionization mode). AD, acteoside degradation product. AD1, unknown; AD2, β-oxoacteoside; AD3, verbasoside; AD4, cistanoside F; AD5, unknown; AD6, unknown; AD7, caffeic acid; AD8, oraposide; AD9, campneoside II; AD10, isocrenatoside; AD11, isocampneoside II; AD12, isoacteoside.
